# Novel Insights into the Regulatory Architecture of CD4+ T Cells in Rheumatoid Arthritis

**DOI:** 10.1371/journal.pone.0100690

**Published:** 2014-06-24

**Authors:** Adrià Aterido, Carlos Palacio, Sara Marsal, Gabriela Ávila, Antonio Julià

**Affiliations:** 1 Rheumatology Research Group, Vall d'Hebron Hospital Research Institute, Barcelona, Spain; 2 Department of Hematology, Hospital Universitari Vall d'Hebron, Barcelona, Spain; University of Kansas Medical Center, United States of America

## Abstract

Rheumatoid arthritis (RA) is the most frequent autoimmune chronic inflammatory disease of the joints and it is characterized by the inflammation of the synovial membrane and the subsequent destruction of the joints. In RA, CD4+ T cells are the main drivers of disease initiation and the perpetuation of the damaging inflammatory process. To date, however, the genetic regulatory mechanisms of CD4+ T cells associated with RA etiology are poorly understood. The genome-wide analysis of expression quantitative trait loci (eQTL) in disease-relevant cell types is a recent genomic integration approach that is providing significant insights into the genetic regulatory mechanisms of many human pathologies. The objective of the present study was to analyze, for the first time, the genome-wide genetic regulatory mechanisms associated with the gene expression of CD4+ T cells in RA. Whole genome gene expression profiling of CD4+ T cells and the genome-wide genotyping (598,258 SNPs) of 29 RA patients with an active disease were performed. In order to avoid the excessive burden of multiple testing associated with genome-wide *trans*-eQTL analysis, we developed and implemented a novel systems genetics approach. Finally, we compared the genomic regulation pattern of CD4+ T cells in RA with the genomic regulation observed in reference lymphoblastoid cell lines (LCLs). We identified a genome-wide significant *cis*-eQTL associated with the expression of *FAM66C* gene (P = 6.51e−9). Using our new systems genetics approach we identified six statistically significant *trans*-eQTLs associated with the expression of *KIAA0101* (P<7.4e−8) and *BIRC5* (P = 5.35e−8) genes. Finally, comparing the genomic regulation profiles between RA CD4+ T cells and control LCLs we found 20 genes showing differential regulatory patterns between both cell types. The present genome-wide eQTL analysis has identified new genetic regulatory elements that are key to the activity of CD4+ T cells in RA.

## Introduction

Rheumatoid arthritis (RA) is the most frequent autoimmune chronic inflammatory disease of the joints and affects up to 1% of the World population. RA is characterized by synovial membrane hyperplasia, increased vascularity and chronic immune cell infiltration that lead to joint destruction and pain [1]. The predominant immune cell type that infiltrates RA synovial joints is the CD4+ T lymphocyte and, for many years, it has been considered a T-cell driven disease [2]. Consequently, treatments targeting the activation of CD4+ T cells have proven successful in the control of disease activity in RA [3], [4]. This evidence, together with the strong genetic association of the molecules that mediate antigen presentation to CD4+ T cells in RA, clearly indicate that the characterization of the regulatory elements of this cell type will be key to completely understand the disease pathogenesis [5]. To date, however, a global analysis of the regulatory mechanisms of CD4+ T cells in RA has not been performed.

Genome-wide association studies (GWAS), in which common genetic variants are tested for association with complex traits, have revolutionized the identification of genetic risk factors for many common diseases [6]. More recently, the integration of GWAS with gene expression data to identify quantitative trait loci (eQTLs) is starting to provide significant insights into the genetic architecture of human diseases [7]. The number of transcripts expressed by a gene is modified by the variation in genetic regulatory elements. RNA levels can therefore be considered as a quantitative trait and used to map these crucial regulatory elements in the genome [8]. Gene expression microarrays and more recently ultra-high throughput RNA sequencing systems coupled with genome-wide genotyping assays are allowing to scan the whole genome variation to identify trait-specific eQTLs [9]. eQTL studies are leading to the characterization of functional sequence variation as well as the understanding of basic genetic regulatory mechanisms [10]. So far, one of the most important discoveries of genome-wide eQTL mapping has been the finding that a substantial fraction of the gene expression regulation is cell type-specific [11]. Consequently, the understanding of the genomic regulatory basis that underlies a complex disease like RA will only be possible if it is performed at the cell type level, in particular analyzing those cell types that play a crucial role in the disease onset and progression [12], [13].

Recently, studies have been reported that characterize regulatory variants that operate in a cell type-specific manner [11], [14], [15]. However, most of these studies focused only on *cis*-acting elements rather than identifying *trans*-acting elements. While c*is*-eQTLs are likely to influence molecular mechanisms involved in transcription, splicing or mRNA decay [16], *trans*-eQTLs are more likely to perturb entire pathways and mediate complex epistatic and gene-environment interactions and, therefore, are of particular interest in the study of prevalent diseases with a complex genetic basis like RA [17], [18]. Importantly, while *cis*-eQTLs are often conserved among different cell types, *trans*-eQTLs tend to be cell type-specific [19]. The genome-wide analysis of *trans*-eQTLs, however, requires the analysis of an exponential number of transcript-SNP pairs, resulting in a prohibitive multiple testing problem. As a consequence, very few studies have explored the presence of *trans*-eQTLs in human traits [20], and new strategies to reduce the burden of multiple testing in these studies must be devised.

In this study, we have analyzed, for the first time, the regulatory variation associated with the gene expression of CD4+ T cells in RA. To do so, we have performed a genome-wide *cis*-eQTL analysis and we have implemented a robust systems genetic approach to perform the *trans*-eQTL analysis. In this approach we exploit the presence of cell-specific gene expression networks, together with the power of biological knowledge and the statistical analysis of networks, to efficiently reduce the high dimensionality associated with the global analysis of *trans*-eQTLs. Finally, in order to identify additional specific gene expression regulation, we have compared the regulatory patterns observed in CD4+ T cells to those of lymphoblastoid cell lines (LCLs), a well-characterized reference cell type. Taken together, the results of this study provide new insights into the key regulatory elements of CD4+ T cells in RA.

## Materials and Methods

### Patients and samples

A total of 29 patients with rheumatoid arthritis from the Rheumatology Unit of the Vall d'Hebron University Hospital (Barcelona, Spain) were recruited. All patients had been diagnosed as RA following the 1987 *American College Rheumatology* criteria [21]. In order to obtain a gene expression profile more representative of the disease, all patients had to have a high disease activity at the moment of sample collection. In this study, high disease activity was defined as an European League against Rheumatoid Arthritis (EULAR) Disease Activity Score (DAS28) [22] higher than 3.2. The DAS28 score efficiently reflects the disease activity of the RA patient by combining the evidence of tenderness and swelling in 28 joints together with the patient's global assessment and a systemic marker of inflammation (erythrocyte sedimentation rate or C-reactive protein levels). In order to avoid the influence of treatment over the gene expression patterns in RA, all patients were receiving the same treatment (≤20 mg/wk metothrexate) and were all naïve to biological immunomodulating agents like anti-TNF agents. Patients suspected to have a concomitant infection or were positive for hepatitis B or C viruses (active or inactive) were also not included in this study. The main features of the RA patient cohort used in this study are shown in Table S1. From each patient, 30 mL of venous blood was obtained, from which 5 mL were used for genomic DNA isolation and 25 mL for CD4+ T cell RNA isolation. Genomic DNA was isolated using the Chemagic Magnetic Separation Module I (PerkinElmer, USA). In order to obtain the total RNA from CD4+ T cells, we first isolated the CD4+ lymphocytes from whole blood using the *RossetteSep* negative selection kit (Stem Cell Technologies, Canada). Isolated cells were immediately preserved in RNA stabilization reagent RNAlater (Qiagen, Hilden, Germany) and frozen at −80°C. In order to determine the level of cell purity, FACS flow cytometry analysis was performed on CD4+ T cells. The CD45+, CD4+, CD3+ and CD8+ T cells were stained by direct immune fluorescence using monoclonal antibodies conjugated with fluorochromes fluorescein isothiocyanate, phycoerythrin, pycoerythrin-cyanin-5 and Phycoerythrin-Texas (all antibodies from Beckman Coulter, FL, USA), respectively. Isotype-matched immunoglobulins with no reactivity against surface markers and the fluorochrome combination were used as negative controls to determine fluorescence background. Acquisition of flow data was performed using an EPICS-XL^-^MCL cytometer and Expo32 software (Beckman-Coulter, FL, USA) after antibody incubation followed by erythrocyte lysis. This analysis was carried out on the same day of blood extraction in all RA patients and >95% CD4+ T cell purity from all samples was confirmed. RNA extraction from the isolated CD4+ T cells was performed with the *RNeasy* extraction kit (Qiagen, Hilden, Germany) and its quality determined using the *2100 BioAnalyzer* system (Agilent technologies, Waldbronn, Germany).

All the procedures followed were in compliance with the principles of the Declaration of Helsinki. All patients provided written informed consent. The study and the consent procedure were approved by the Institut de Recerca Hospital Universitari Vall d'Hebron ethics committee.

### Gene expression profiling

Whole genome transcript abundance from the CD4+ T cells of patients with RA was performed using the Illumina Human-6 v1 Beadchip array system (Illumina, San Diego, CA, USA). This microarray platform measures the gene expression levels of more than 47,000 different transcripts. In order to update the probe annotation for this microarray, we used the NCBI RefSeq database [23]. Matching the microarray probe sequence to the latest RefSeq database version (2^nd^ May 2013) we found that 13,555 probes perfectly mapped to unique transcripts, 26,729 probes that did not map any transcript and 8,013 probes that mapped more than one transcript from which 7,565 mapped to the same gene. Consequently, the updated microarray probe annotation was composed by 21,120 probes matching to known transcripts. Data preprocessing was conducted using the R statistical software [24]. The raw expression intensities of the 29 microarrays were processed using background adjustment. One sample showed intensity dependent biases and it was finally removed. The gene expression intensities were normalized on the log2-scale using the quantile normalization method [25]. In order to remove the potential variability introduced by the presence of different microarray processing batches, we used the ComBat empirical Bayes method [26]. The data discussed in this publication have been deposited in NCBI's Gene Expression Omnibus [27] and are accessible through GEO Series accession number GSE55468.

### Whole genome genotyping

The genome-wide genotyping of the 29 RA patients was performed using the Illumina Quad610 Beadchips (Illumina, San Diego, California, USA). The Quad610 genotyping arrays scan 618,150 polymorphisms (598,258 SNPs and 19,892 CNV probes). Genotype calling was performed using the GenomeStudio data analysis software (v2011.1, Illumina, San Diego, California, USA). The quality control evaluation was performed using PLINK software [28]. All the autosomal SNPs were initially selected (n = 582,539). A total of 7,886 markers showing >10% missing data were excluded, as well as 1,724 SNPs with P<1e-5 for test of deviance from Hardy-Weinberg equilibrium. After all quality control steps, a final number of 572,980 SNPs were used for the eQTL analysis in CD4+ T cells. In order to compare the exact same polymorphisms in the CD4+ T cell and LCL eQTL analyses, 6,962 SNPs had to be excluded from the initial dataset. In this case, a final number of 566,018 SNPs were used for the comparative eQTL analysis between both cell types. The presence of population stratification in the study samples was estimated using principal component analysis (PCA) implemented in EIGENSOFT (v4.2) software [29]. Using the top ten principal components over ten iterations and using a threshold of six standard deviations, we excluded two samples showing an outlier genetic background.

### Statistical association analysis

*Cis-* and *trans*-eQTL analyses were performed using Matrix eQTL software [30]. Matrix eQTL efficiently performs large numbers of eQTL analyses by the use of large matrix multiplications. Most gene regulatory elements that act in *cis* have been previously reported to be located in close proximity to the gene [31]. However, there is clear evidence that *cis* regulatory elements like enhancers can be located as far as 1 Mb from the gene they regulate [32]. Consequently, we used 1 Mb as the maximal distance at which *cis* regulation can occur. This distance is in accordance to most recent studies on genome-wide eQTLs [10]. For each transcript-SNP pair, we fitted the following linear model assuming additive effect of genotype on gene expression:
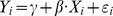



The genotype *X_i_* of individual *i* at given SNP is encoded by 0, 1 and 2 according to the number of minor alleles present in the genotype of the individual. The gene expression *Y_i_* is the normalized log-expression level of the probe for individual *i*. The *ε_i_* captures all other factors which influence the gene expression. The null hypothesis of the statistical test was that there is no association between the genotype and the gene expression (*β = 0*). In the present study, we included gender as a covariate. In order to avoid false positives due to low allele frequency, we filtered those SNPs with a minor allele frequency <10%. Multiple testing correction was performed using the False Discovery Rate (FDR) method [33]. A total of 21,120 transcripts and 572,980 SNPs measured in 26 individuals were finally used for the *cis*-eQTL analysis in CD4+ T cells.

### Novel systems genetics approach for *trans*-eQTL identification

**Figure 1:**
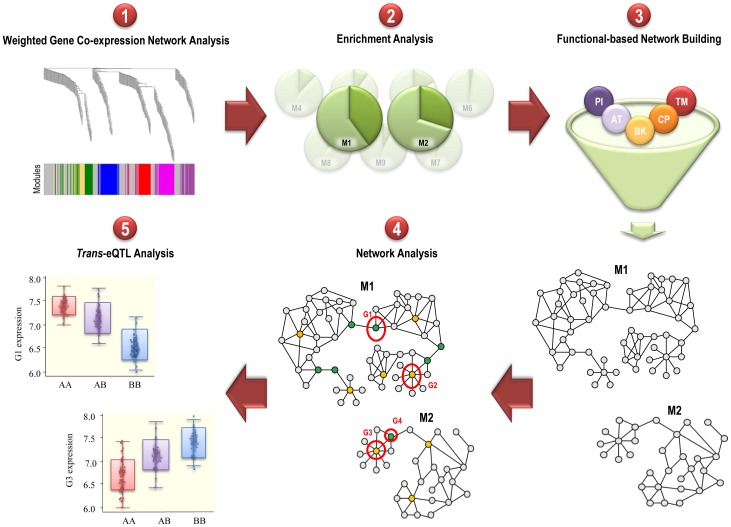
Workflow of the systems genetics approach for *trans*-eQTL identification. The new systems genetics approach is based on four steps that are performed before the *trans*-eQTL analysis in order to efficiently reduce the number of analyzed genes: 1) Identification of the gene expression modules (M) that characterize CD4+ T cell gene expression 2) Enrichment analysis of a specific biological process that is related to the trait of interest 3) Construction of the functional-based networks using biological knowledge within the significantly enriched modules 4) Network analysis to identify those genes that are likely to play a central role in the functional-based networks 5) *Trans*-eQTL analysis using the subset of genes that show the highest centrality in each module. Abbreviations: AT, association transfer between organisms; BK, curated biological pathway knowledge; CP, computational predictions; G, gene; M, module; PI, physical interactions; TM, text-mining.

We present a novel systems genetics approach to address the dimensionality problem present in *trans*-eQTL analyses. The pipeline of this approach is composed by five consecutive steps (Figure 1).

In the first step, the gene expression modules that characterize the cell type of interest are identified using the genome-wide expression data. There are several methods that can be used for this objective. In our study, we used the weighted correlation network analysis implemented in the WGCNA R software package [34]. In this method, the correlation between genes is used to compute a network adjacency matrix, which fully determines the gene co-expression networks. From this network adjacency matrix, WGCNA then uses an unsupervised clustering approach to identify the gene expression modules that best characterize the gene expression of the cell or tissue of interest.

In the second step, the gene expression modules that characterize the cell type of interest are analyzed for enrichment of a specific biological process that is related to the disease or trait of interest (i.e. cell cycle in cancer studies). For this objective, a biological annotation database like the gene ontology (GO) database [35] is used. The enrichment can then be quantitatively measured using well-known statistical methods. In our study, we assessed the statistical significance of the GO functional enrichment using the Fisher's exact test as described previously [36]. Only those gene expression modules that are significantly enriched by the biological process of interest are selected for the next step.

In the third step, the biological knowledge is used to build functional-based networks from the gene expression modules of interest. In these functional-based networks, the nodes are the genes from the selected gene expression modules. Then, according to the presence or absence of biological evidence between each gene pair, the edges of the network are established. For this objective, a complete and updated database of functional associations is required. In our study, we used STRING (v9.1, 2013) software tool [37]. STRING is a powerful bioinformatics database that integrates five different sources of functional associations between genes in more than 1,100 organisms. These sources are i) physical interactions, ii) curated biological pathway knowledge, iii) computational predictions, iv) text-mining and v) association transfer between organisms. The association transfer between organisms is based on the principle of interaction conservation, which means that a pair of proteins binding in one organism is expected to be binding in another organism if both genes are present in both genomes. Therefore, the functional associations in one organism can be transferred to another organism using comparative genomics.

In the fourth step of this approach, network analysis methods are used to identify those genes that are likely to play a central role in the previously identified functional-based networks. In network analysis, the most influential genes are those that show either many connections to other genes and/or that exert an essential connection between gene (node) subnetworks. These two features are commonly known as degree centrality (DC) and betweenness centrality (BC), respectively. Genes with high DC are defined as hubs and may also be more evolutionary conserved than non-hubs [38]. Bottlenecks, that is genes with high BC, are more likely to be essential than proteins with low BC [39], [40]. In our study, the network analysis was performed using the Cytoscape v3.0.1 software [41].

In the last step of this systems genetics method, only those genes that are more likely to play a central role in the cell-specific network are selected and analyzed for the *trans*-eQTL analysis. These genes are selected according to their DC and BC values obtained in the previous step and, therefore, will have a higher probability to be associated to an influential genetic variation in the cell type of interest.

With this systems genetics approach, biological and network information is efficiently used to objectively reduce the number of genes included in the *trans*-eQTL analysis to those with highest influence on the cell type of interest and, therefore, increase the likelihood of identifying relevant *trans*-eQTL associations.

### Analysis of differential genomic regulation profiles

In order to compare the genomic regulation profiles of CD4+ T cells and LCL cells, we focused on the study of *cis*- and *trans*-eQTL associations of two different groups of genes. In the first group, we analyzed those genes previously associated with RA risk and belonging to known biological pathways [42]. In the second group, we analyzed those genes associated with the T cell differentiation into different CD4+ T cell subtypes, including T helper 1 (Th1), T helper 2 (Th2), T helper 17 (Th17) and T regulatory (Treg) subtypes. This last set of genes was extracted from the Kyoto Encyclopedia of Genes and Genomes (KEGG) database [43]. In total, the comparative eQTL analysis between CD4+ T cells and LCL cells was performed using 145 transcripts, corresponding to 113 different genes (Table S2). The LCL gene expression profiles and the corresponding whole genome genotype data were obtained from 26 unrelated Caucasian European (CEU) individuals from the HapMap reference project [44].

In order to avoid the study of redundant eQTLs (i.e. neighboring SNPs in high linkage disequilibrium associated to one single transcript), we divided the autosomal chromosomes into 32,962 independent loci according to the localization of high recombination sites (i.e. *hotspots*) [45]. The whole set of SNPs were mapped to these independent loci in order to be able to determine the Transcript Complexity Value (TCV). The TCV represents the number of independent loci that are associated to the expression of one particular gene. Using the *cis*- (P<0.05) and *trans*-eQTLs (P<1e−5) identified in the CD4+ T cells and LCLs analyses, we computed the TCV for each of the 113 selected genes. The statistical significance of the differences in TCVs between both cell types was assessed using the Fisher's exact test.

## Results

### Genome-wide *cis*-eQTL analysis in RA CD4+ T cells

After performing the *cis*-eQTL analysis of the gene expression of CD4+ T cell from RA patients having an active disease (n = 8,747,394 tests), we detected two genome-wide significant associations with *FAM66C* gene: SNP rs7976243 (chromosome 12p13.3, P_FDR_ = 2.85e−2) and SNP rs2244822 (chromosome 12p13.3, P_FDR_ = 2.85e−2). Both genome-wide significant *cis*-eQTLs are only 2.14 Kb apart and, as expected, they are in high linkage disequilibrium (r^2^ = 0.95, HapMap Caucasian European samples) and consequently, they represent the same association signal. A complete list of c*is*-eQTLs from RA CD4+ T cells having a P_FDR_<0.5 is shown in Table S3.

### *Trans*-eQTL analysis in RA CD4+ T cells using the novel systems genetics approach

**Figure 2:**
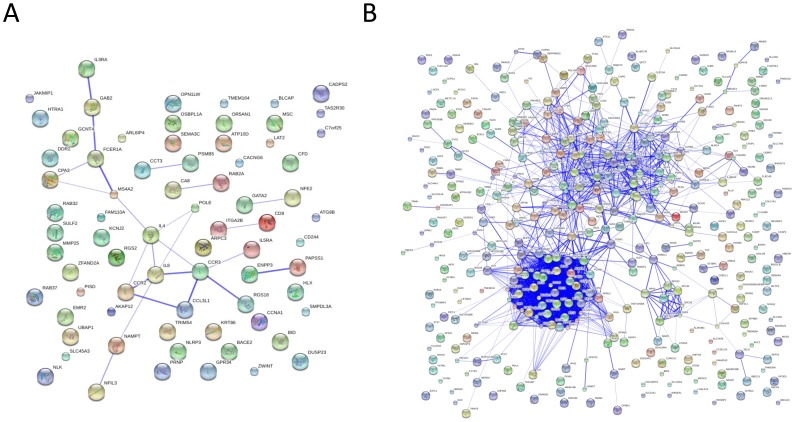
Building of functional-based networks for M9 and M12 CD4+ gene expression modules using biological knowledge. **A**: Functional-based network obtained from the immunologically enriched M9 gene expression module where 15 genes display a connected network. **B**: Functional-based network obtained from the immunologically enriched M12 gene expression module, which shows a complex network structure with different subnetworks involving 247 interconnected genes.

A total of 16 gene expression modules were found to characterize the CD4+ T cell gene expression of RA patients with active disease (Table 1). Given that RA is an autoimmune disease characterized by the chronic activity of inflammatory cells, we performed the functional enrichment analysis of the CD4+ T cell modules over GO terms related to the immune system. From the 16 gene expression modules identified in RA CD4+ T cells, we found two modules to be highly significantly enriched in genes related to the immune system (P = 9.23e−21 and P = 9.14e−5 for modules 9 and 12, respectively). Using these two immune-related gene expression modules, we built their corresponding functional-based networks. The M9 CD4+ T cell module functional analysis revealed a network of 15 interconnected genes (Figure 2 A). The analysis of the M12 gene-expression module showed larger and more complex network involving 247 genes. The topological structure of the M12 functional network (Figure 2 B) suggests the existence of different subnetworks that are connected by a few genes (i.e. genes showing high BC).

**Table 1:** Gene expression modules identified in RA CD4+ T cells.

Module	Number of Transcripts	Immune System Process	P-value
M1	288	NS	3.69e-01
M2	2326	Underrepresented	5.00e-03*
M3	1682	Underrepresented	3.41e-06*
M4	83	Underrepresented	1.26e-02*
M5	755	NS	2.64e-01
M6	155	NS	3.69e-01
M7	10003	NS	7.71e-01
M8	199	NS	4.94e-01
M9	75	Overrepresented	9.23e-21*
M10	253	NS	1.36e-01
M11	157	NS	7.50e-01
M12	402	Overrepresented	9.14e-05*
M13	124	Underrepresented	5.34e-23*
M14	136	Underrepresented	2.82e-06*
M15	2904	NS	7.90e-02
M16	1578	Underrepresented	3.50e-05*

Applying the new systems genetics approach for *trans*-eQTL identification in RA CD4+ T cells and using WGCNA, 16 genes expression modules (M) that characterize the CD4+ T cells were identified. For each gene expression module, the number of gene transcripts representing each module is shown, as well as the results of the immunological enrichment analysis.

Abbreviations: NS, No Significant.

* Significant (P<0.05).

**Figure 3:**
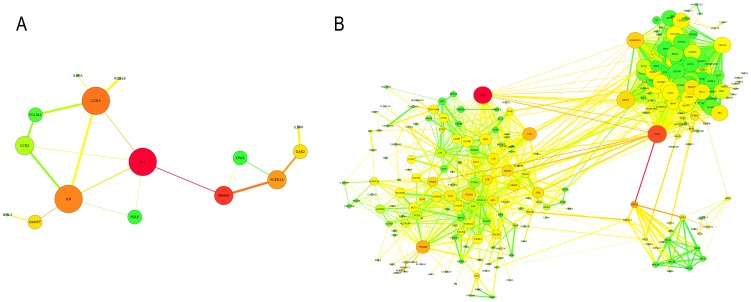
Functional-based networks analyzed in each enriched CD4+ T cell gene expression module. **A**: Functional-based network obtained from the immunologically enriched M9 gene expression module. **B**: Functional-based network obtained from the immunologically enriched M12 gene expression module. The dimensions of each node (i.e. gene) are proportional to its DC value and its color is based on its BC value, ranging from green (lowest BC values) to red (highest BC values). The edge width is proportional to the strength of the functional association evidences between two genes. The edge betweenness determines the edge color, ranging from green (edges connecting nodes with the lowest BC values) to red (edges connecting nodes with the highest BC values).

After computing the network properties of each gene in the identified M9 and M12 functional networks (Table 2), we selected those genes showing the highest centrality measures. A total of 13 genes showing either high connectivity with other genes (DC_M9_≥5, DC_M12_≥51) or high connectivity between node subnetworks (BC_M9_≥0.26, BC_M12_≥0.05) were selected. These selected genes were *IL4*, *MS4A2*, *CCR3*, *IL8* and *FCER1A* genes from M9 module and *TSPO*, *CDK1*, *RPS3*, *TYROBP*, *CD4, BIRC5, CDC45 and KIAA0101* genes from M12 module (Figure 3). Using this set of genes that have a very high probability of being relevant in the CD4+ T cell pathophysiology in RA, we finally performed the *trans*-eQTL analysis (N_M9_ = 3,435,556 tests, N_M12_ = 5,725,245 tests). After multiple test correction, we identified six statistically significant *trans*-eQTLs (Table 3). A complete list of the *trans*-eQTLs having a nominal P-value<1e-5 in modules M9 and M12 is shown in Tables S4 and S5, respectively.

**Table 2:** Network statistics of M9 and M12 RA CD4+ gene expression modules.

Module	Gene Symbol	BC	DC	Module	Gene Symbol	DC	BC
M9	*IL4*	0.5329	5	M9	*IL4*	5	0.5329
M9	*MS4A2*	0.4395	3	M9	*CCR3*	5	0.3297
M9	*CCR3*	0.3297	5	M9	*IL8*	5	0.3021
M9	*IL8*	0.3021	5	M9	*MS4A2*	3	0.4395
M9	*FCER1A*	0.2637	3	M9	*FCER1A*	3	0.2637
M9	*NAMPT*	0.1429	2	M9	*CCR2*	3	0.0549
M9	*GAB2*	0.1429	2	M9	*NAMPT*	2	0.1429
M9	*CCR2*	0.0549	3	M9	*GAB2*	2	0.1429
M9	*CCL3L1*	0.0110	2	M9	*CCL3L1*	2	0.0110
M9	*RGS18*	0.0000	1	M9	*POLE*	2	0.0000
M12	*TSPO*	0.2436	55	M12	*CDK1*	57	0.1717
M12	*CDK1*	0.1717	57	M12	*TSPO*	55	0.2436
M12	*RPS3*	0.0905	11	M12	*BIRC5*	53	0.0432
M12	*TYROBP*	0.0836	35	M12	*CDC45*	51	0.0138
M12	*CD4*	0.0738	36	M12	*KIAA0101*	50	0.0515
M12	*KIAA0101*	0.0515	50	M12	*TOP2A*	50	0.0156
M12	*MNDA*	0.0472	20	M12	*CCNA2*	50	0.0036
M12	*MAPK1*	0.0455	25	M12	*TK1*	49	0.0246
M12	*BIRC5*	0.0432	53	M12	*CDT1*	49	0.0156
M12	*ITGAM*	0.0425	34	M12	*DLGAP5*	49	0.0103

Applying the new systems genetics approach for *trans*-eQTL identification in RA CD4+ T cells, the BC and DC values were computed for each particular gene of M9 and M12 immunity-enriched modules. On the left side of the table, the top 10 genes showing the highest BC values in each module are sorted by BC in decreasing order. On the right side of the table, the top 10 genes showing the highest DC values in each module are sorted by DC in decreasing order.

Abbreviations: BC, Betweenness centrality; DC, Degree centrality.

**Table 3:** *Trans*-eQTL associations revealed by the application of the systems genetics approach.

Module	SNP	Gene	SNP Coordinates (bp)	Gene Coordinates (bp)	β	P-value	P_FDR_
M9	rs9293162	*FCER1A*	chr5:25065470	chr1:159253678–159278014	1,851	1.1927e-07	0.077
M9	rs978897	*FCER1A*	chr7:15598156	chr1:159253678–159278014	1,670	3.2865e-07	0.094
M12	rs3862556	*KIAA0101*	chr10:76540987	chr15:64657210–64673702	−0,924	2.4332e-08	0.046*
M12	rs711114	*KIAA0101*	chr12:78003541	chr15:64657210–64673702	−0,848	5.0807e-08	0.048*
M12	rs10283761	*BIRC5*	chr9:26755930	chr17:76210276–76221716	−0,345	5.3493e-08	0.048*
M12	rs9561023	*KIAA0101*	chr13:93022401	chr15:64657210–64673702	−0,999	6.0208e-08	0.048*
M12	rs2513046	*KIAA0101*	chr11:62236094	chr15:64657210–64673702	−0,999	6.0208e-08	0.048*
M12	rs17009383	*KIAA0101*	chr3:21771769	chr15:64657210–64673702	−0,573	7.3813e-08	0.048*
M12	rs4806933	*KIAA0101*	chr19:3414020	chr15:64657210–64673702	−0,727	1.6853e-07	0.079
M12	rs4745758	*KIAA0101*	chr10:76568257	chr15:64657210–64673702	−0,814	4.7989e-07	0.095
M12	rs11001178	*KIAA0101*	chr10:76601805	chr15:64657210–64673702	−0,814	4.7989e-07	0.095
M12	rs10824245	*KIAA0101*	chr10:76666799	chr15:64657210–64673702	−0,814	4.7989e-07	0.095
M12	rs736086	*KIAA0101*	chr10:76683785	chr15:64657210–64673702	−0,814	4.7989e-07	0.095
M12	rs4746248	*KIAA0101*	chr10:76705881	chr15:64657210–64673702	−0,814	4.7989e-07	0.095
M12	rs7046685	*KIAA0101*	chr9:28659143	chr15:64657210–64673702	−0,873	5.0499e-07	0.095
M12	rs12341535	*KIAA0101*	chr9:28663042	chr15:64657210–64673702	−0,873	5.0499e-07	0.095
M12	rs17101861	*KIAA0101*	chr14:34232772	chr15:64657210–64673702	−0,873	5.0499e-07	0.095
M12	rs6756606	*CDK1*	chr2:178700057	chr10:62538211–62554610	−0,688	5.2009e-07	0.095
M12	rs10497482	*CDK1*	chr2:178805428	chr10:62538211–62554610	−0,688	5.2009e-07	0.095
M12	rs6756606	*KIAA0101*	chr2:178700057	chr15:64657210–64673702	−0,955	5.5269e-07	0.095
M12	rs10497482	*KIAA0101*	chr2:178805428	chr15:64657210–64673702	−0,955	5.5269e-07	0.095
M12	rs2714384	*BIRC5*	chr18:25302948	chr17:76210276–76221716	−0,348	6.4318e-07	0.098
M12	rs2617950	*BIRC5*	chr18:25309414	chr17:76210276–76221716	−0,348	6.4318e-07	0.098
M12	rs2851757	*BIRC5*	chr18:25313101	chr17:76210276–76221716	−0,348	6.4318e-07	0.098

Applying the new systems genetics approach for *trans*-eQTL identification in RA CD4+ T cells, the associations obtained after performing the *trans*-eQTL analysis are displayed for each immunity-enriched module (P_FDR_<0.01).

Abbreviations: Bp, Base pair; β, Slope coefficient; P_FDR_, P-value False Discovery Rate.

* Significant (P_FDR_<0.05).

The statistically significant *trans*-eQTLs identified with our novel systems genetics approach were associated to the expression of *KIAA0101* and *BIRC5* genes, both central genes of the M12 CD4+ T cell network. Five of the significant *trans*-eQTLs were associated to the expression of *KIAA0101* and are located in different chromosomic regions: SNP rs3862556 (intergenic variant, chromosome 10q22.2, P_FDR_ = 4.6e−2), SNPS rs711114 (intergenic variant, chromosome 12q21.1, P_FDR_ = 4.8e−2), SNP rs9561023 (*GPC5* locus, chromosome 13q31.3, P_FDR_ = 4.8e−2), SNP rs2513046 (*AHNAK* locus, chromosome 11q12.1, P_FDR_ = 4.8e−2) and SNP rs17009383 (*ZNF385D* locus, chromosome 3p24.3, P_FDR_ = 4.8e−2). The remaining *trans*-eQTL was established between the genetic variation at SNP rs10283761 (intergenic variant, chromosome 9p21.2, P_FDR_ = 4.8e-2) and the expression levels of the *BIRC5* gene.

### Analysis of differential genomic regulation profiles

The comparison of the genomic regulation profiles of CD4+ T cells and LCL cells revealed several genes showing significantly different genetic regulatory mechanisms between both cell types.

**Figure 4:**
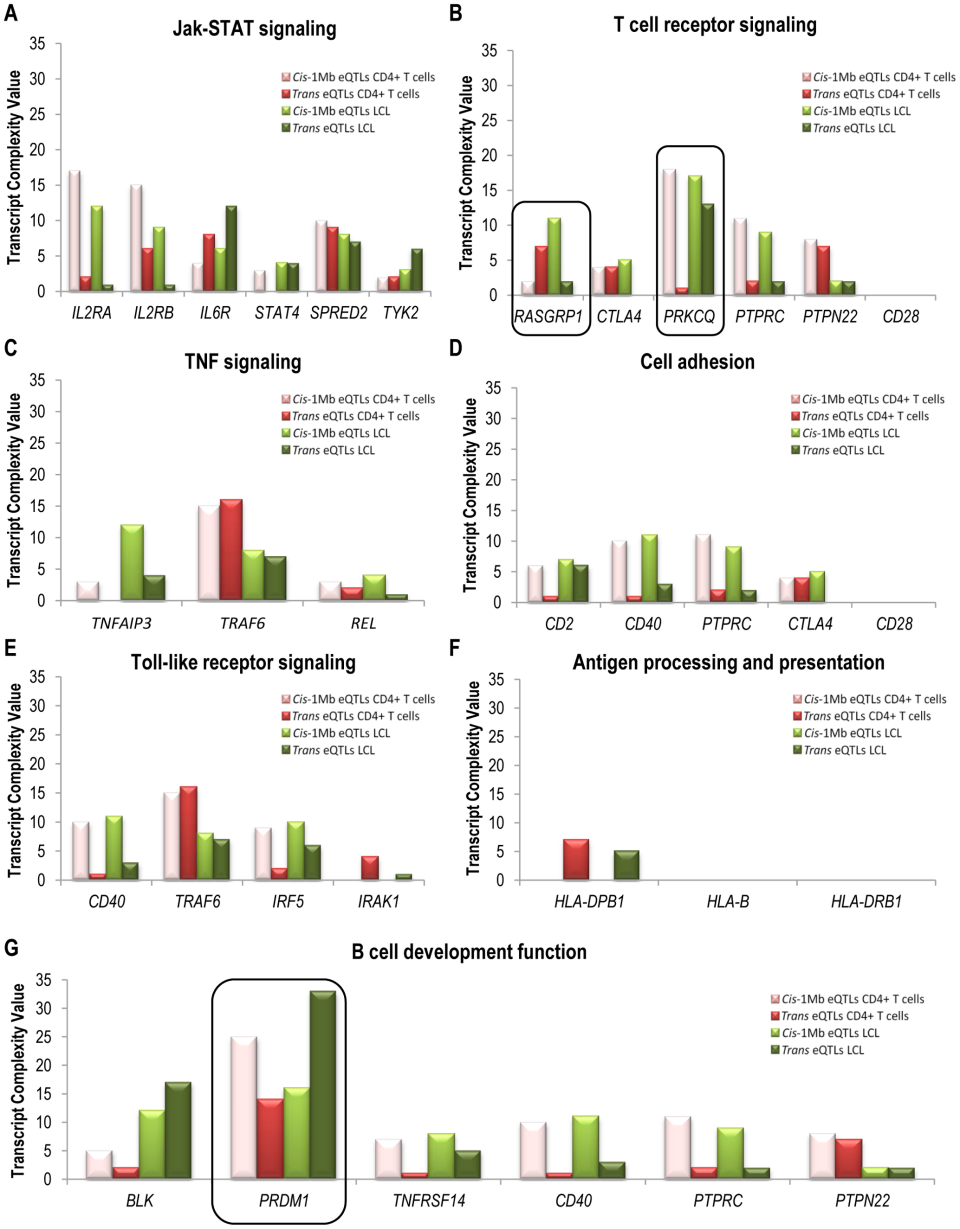
Transcript Complexity Value analysis of RA risk genes. TCV from the RA CD4+ T cell and control LCLs analyzed in RA risk genes. **A**: JAK-STAT signaling pathway. **B**: T cell receptor signaling pathway. **C**: TNF signaling pathway. **D**: Cell adhesion pathway. **E**: Toll-like receptor signaling pathway. **F**: Antigen processing and presentation pathway. **G**: B cell development function pathway. The genes that are framed represent those genes showing significant genomic regulation profiles between RA CD4+ T cells and LCLs (P<0.05).

In the RA risk gene group, three genes showing a highly differential genomic regulation between CD4+ T cells and LCLs were identified (Figure 4). In the T cell receptor signaling pathway, *PRKCQ* gene had similar TCVs in LCLs *cis*- and *trans*-eQTLs while CD4+ T cells had a high *cis*-TCV and a practically absent *trans*-TCV (P = 4.1e−3). In this same pathway, *RASGRP1* gene showed an opposite regulatory pattern in both cell types, with a predominant *trans*-TCV in CD4+ T cells and a predominant *cis*-TCV in LCLs (P = 7.3e−3). Finally, *PRDM1* gene, belonging to the B cell development pathway, was found to be mainly *cis*-regulated in CD4+ T cells and *trans*-regulated in LCLs (P = 4.9e−3).

**Figure 5:**
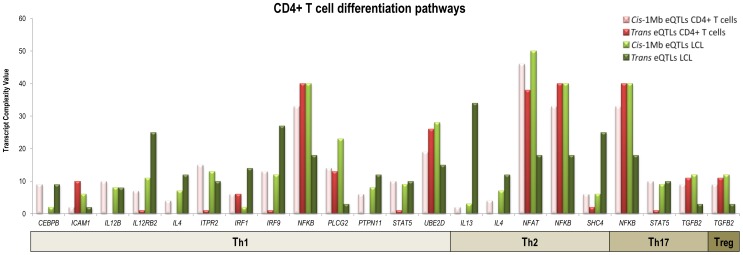
Transcript Complexity Value analysis of T cell differentiation genes. The type and quantity of significant eQTLs in RA CD4+ T cells and LCL cells are compared in the genes from the Th1, Th2, Th17 and Treg differentiation pathways. Only those genes showing a significantly different genomic regulation profile (P<0.05) are shown.

Among those genes involved in T cell differentiation pathways, we found a total of 17 genes showing a differential regulatory profile between both cell types. Taking into account that CD4+ T cell differentiation pathways can share several genes, we found a differential regulation in 13, 5, 3 and 1 genes from the Th1, Th2, Th17 and Treg differentiation pathways, respectively (Figure 5).

*NFKB* (P = 8.0e−3), *ICAM1* (P = 1.9e−2), *UBE2D* (P = 3.5e−2) and *TGFB2* (P = 4.6e−2) genes showed opposite regulatory profiles between both cell types, with a higher contribution of *trans* regulation in CD4+ T cells and a higher contribution of *cis* regulation in LCLs. Conversely, we found that the expression of *IRF9* (P = 7.9e−6), *IL12RB2* (P = 4.9e−3), *SHC4* (P = 5.6e−3), *IL12B* (P = 9.5e−3), *IL13* (P = 1.3e−2), *ITPR2* (P = 1.4e−2), *PTPN11* (P = 1.7e−2), *STAT5* (P = 2.3e−2) and *IL4* (P = 3.7e−2) genes were predominantly regulated by *cis*-acting elements in CD4+ T cells from RA patients.

The *IRF1* gene expression, whose *cis*- and *trans*- regulation was clearly found to be similar in RA CD4+ T cells, showed a markedly higher *trans*-eQTL regulation and almost absent *cis*-eQTL evidence of regulation in LCLs (P = 4.4e−2). *PLCG2* gene expression regulation was also similar in CD4+ T cells, but had a markedly high *cis* regulation and almost absent *trans* regulation in LCLs (P = 6.2e−3). Finally, the regulatory profile of *CEBPB* gene expression was characterized by the absence of a *trans* regulation in RA CD4+ T cells and the clear effect of *trans*-acting regulatory elements in LCLs (P = 3.3e−4).

The complete list of c*is*- and *trans*-eQTLs in CD4+ T cells and LCLs that were used to characterize these regulatory profiles are shown in Table S6, Table S7, Table S8 and Table S9.

## Discussion

In the present study we have performed a genome-wide analysis of eQTLs in CD4+ T cells and we have identified new genetic regulatory variants associated with the gene expression of this key cell type in RA. Analyzing the transcriptome of CD4+ T cells from RA patients with active disease we found a genome-wide significant *cis*-eQTL regulating the expression of *FAM66C* gene. In order to detect significant *trans*-eQTLs, we developed a novel systems genetics approach that integrates gene expression and network biology information to reduce the multiple test burden associated with this type of analysis. Using this new approach, we found statistically significant *trans*-eQTLs regulating the expression of *BIRC5* and *KIAA0101* genes in CD4+ T cells in RA. Finally, comparing the genetic regulatory patterns of RA CD4+ T cells with control LCLs, we found several differential regulatory patterns. This study represents the first global analysis of the CD4+ T cell regulatory architecture associated with RA.

*FAM66C* is a long non-coding RNA (lncRNA) gene, whose biological functionality is still unknown. LncRNAs genes are transcribed into non-protein coding transcripts that are longer than 200 nucleotides and have been shown to act as modulators of the gene expression through epigenetic changes, transcriptional regulation and post-transcriptional regulation [46]. To our knowledge, the *FAM66C* lncRNA gene has not been previously associated with the pathophysiology of RA or any other complex disease. Recently, lncRNAs have emerged as possible contributors to the basis of human diseases by regulating the expression of neighbouring protein-coding genes [46], [47]. Interestingly, *FAM66C* lncRNA gene maps near *C3AR1* gene (<115 Kb), which encodes a complement receptor that has been shown to be crucial in the modulation of the function of CD4+ T cell subtypes [48]. *C3AR1* expression promotes the proinflammatory activity of CD4+ T cells by enhancing the survival and function of Th1 and Th17 cells, while its inhibition leads to the induction of Treg CD4+ cells [49]. Additional studies will need to be carried out to determine if this or other biological mechanisms are responsible for the observed *FAM66C* association in the RA CD4+ T cell transcriptome.

Genes that encode proteins with high DC and BC values are likely to have a high impact in the network functionality. Consequently, the characterization of such central genes in the functional networks of specific cell types can be a powerful strategy to identify regulatory variants that contribute to disease [50], [51]. Based on this assumption we developed a new dimensionality reduction approach that allowed us to determine the most influential genes in the CD4+ T cell specific networks and identify significant *trans*-eQTL associations with two of these genes, *BIRC5* and *KIAA0101*. These cell type-specific regulatory mechanisms are therefore likely to be of high importance in the activity of CD4+ T cells associated with RA pathology.

*BIRC5* gene encodes survivin, an antiapoptotic protein that has been strongly associated to RA pathogenesis [52], [53], [54]. Survivin mRNA levels in peripherial blood mononuclear cells have been significantly associated with disease activity and the extent of joint damage in RA [55]. Importantly, survivin expression has been shown to be a key promoter of T cell proliferation after antigen presentation as well as a powerful antagonizer of apoptosis in activated T cells [56], [57]. Increased proliferation and reduced cell death are two of the main characteristics of the CD4+ T cell population infiltrating the synovial membrane in RA [58], [59]. Survivin-mediated cell survival could therefore have a major role in maintaining this pathogenic CD4+ T cell features. Additionally, there is recent evidence demonstrating that survivin expression in CD4+ T cells is activated by TNF-α cytokine which is the key regulator of the inflammatory and tissue-destructive pathways in RA [56].

*KIAA0101* gene encodes for the Proliferating Cell Nuclear Antigen (PCNA) associated factor that acts as a regulator of DNA repair during DNA replication [60]. Importantly, PCNA-associated factor interacts with PCNA which increases the DNA polymerase's processivity during elongation of the leading strand and, therefore, accelerates the cell cycle progression [61]. In RA, an increased rate of cell proliferation has been shown to be associated with high levels of PCNA in synovial fibroblasts [62]. Accordingly, we suggest that the increased proliferation and reduced apoptosis of CD4+ T cells in the synovial membrane in RA is also regulated by the expression of *KIAA0101* gene.

The main goal of the new systems genetics approach described in this study is to objectively reduce the multiple test problem associated with the genome-wide analysis of *trans*-eQTLs. An exhaustive *trans*-eQTL analysis at a genome-wide scale would have required to perform approximately 12,000e6 association tests. This extremely high number of tests would have lead to an excessive penalization for multiple testing and, consequently, the inability to detect true associations. Using the new systems genetics approach we reduced this number of tests to 3.4e6 and 5.7e6 trans-eQTL analyses for M9 and M12 functional networks, respectively. Clearly, our study demonstrates that this methodological approach can be useful to identify significant *trans*-eQTLs without the need to explore all the combinatorial space. Importantly, the proposed systems genetics workflow includes several steps that can be easily customized to incorporate new or alternative bioinformatics methodologies. For example, in the functional enrichment analysis we used the gene ontology database but other functional annotation databases like the KEGG database can be used instead. The flexibility of this systems genetics approach workflow also makes this method a powerful strategy to uncover the relevant *trans*-eQTLs associated with human traits or diseases, including the upcoming studies based on RNA-seq technologies.

*Trans*-eQTL associations present in CD4+ T cells and absent in LCLs could be indicative of cell-specific regulatory processes that are specifically activated in RA. In the group of genes associated with RA risk, we found *RASGRP1* and *PRDM1* genes to have a differential regulation between both cell types. *RASGRP1* has been shown to be a critical regulator of the ERK/MAP signaling pathway which is crucial for T cell development, homeostasis and differentiation. T cells from patients with RA have been shown to have hyperresponsive ERK activity upon TCR stimulation [63]. Consequently, *RASGRP1* expression could be a specific modulator of the CD4+ T cell hyperresponsiveness to autoantigens associated with RA. *PRDM1* gene, instead, has been shown to drive the maturation of B-lymphocytes into immunoglobulin-secreting cells [64]. Consistently, we found a predominant *trans* regulation of *PRDM1* gene expression in LCLs, which are cell lines originally generated from B cells.

Among those genes involved in the CD4+ T cell differentiation pathways, we found a markedly differential regulatory profile of *Nuclear Factor Kappa B* (*NFKB*) and *Transforming Growth Factor Beta 2* (*TGFB2*) genes. NKFB is a well known transcription factor that has been associated with the T cell differentiation into Th1, Th2 and Th17 subtypes [65] and is a pivotal regulator of the inflammatory process present in rheumatoid arthritis [66], [67], [68]. The identification of the genetic variants that control *NFKB* gene expression in CD4+ T cells could lead to a better understanding of the biological mechanisms that are more relevant in the regulation of this cell type in RA.

TGFB2 is a Transforming Growth Factor family cytokine that has been associated with immunological tolerance and Treg and Th17 pathways [69]. Previous studies have shown that normal Treg/Th17 cell balance is not maintained in RA, with an increase in the differentiation of CD4+ T cells into the proinflammatory CD4+ Th17 phenotype and a decrease in the production of anti-inflammatory CD4+ Tregs [70], [71], [72]. Therefore, the increased *trans*-eQTLs associated with CD4+ T cells compared to LCLs, could indicate a specific regulatory mechanism associated with the increase of T cell autoreactivity observed in RA pathophysiology.

The different methodologies used in this study for the characterization of the CD4+ T cell-specific genetic regulation in RA have nonetheless some limitations. The CD4+ T cell population is highly heterogeneous with different subtypes exerting sometimes opposing regulatory activities in inflammation. Therefore, a more comprehensive analysis would have required the isolation and separate analysis of each CD4+ subpopulation. However, by using a homogeneous cohort of RA patients with a high level of disease activity, we favored the collection of a highly similar gene expression profile representative of the pathogenic regulation of CD4+ T cells in RA. Also, the comparison of the CD4+ regulatory pattern against LCLs of control individuals could have limited the identification of additional relevant regulatory mechanisms. As more eQTL data on different cell subtypes becomes available, more cell-to-cell comparisons can be performed in order to completely characterize the specific regulatory mechanisms of CD4+ T cells in RA.

In the analysis of differential genomic regulation profiles we focused on genes associated with the susceptibility to develop RA as well as genes associated with T cell differentiation. Another potential limitation of this approach is that other genes that encode proteins associated with RA pathophysiology are not included. From these, the pro-inflammatory cytokines TNF-α [73], IL-1β [74] and IFN-γ [75] and the anti-inflammatory cytokine IL-10 [76] have shown to be key in the development and chronification of RA. However, analyzing the regulatory profiles for these genes only a significant differential regulation for IL-1β is observed (data not shown). This association is due to a predominant *cis*-regulation in CD4+ T cells compared to an increased *trans*-regulation in LCLs. This result is consistent with the high level of expression of this cytokine observed in different immune and non-immune cell types like monocytes, tissue macrophages or synovial fibroblasts [77], [78]. Together, the results of our study support the use of this methodology to characterize the functionality of disease risk genes as well as genes annotated to the cell type of interest.

One of the most important challenges ahead in human genetics is to identify the regulatory elements that control the gene expression and how they contribute to disease. This study is the first approach to the characterization of the CD4+ T cell regulatory profile associated with RA. In this comprehensive genetic study we report genetic regulatory variants that are significantly associated with the expression of *FAM66C* lncRNA, *BIRC5* and *KIAA0101* genes in RA CD4+ T cells. These results highlight the importance of the cell cycle processes in the pathological activity of RA CD4+ T cells infiltrating the synovial membrane, as well as the potential implication of lncRNA in the genetic regulatory basis of RA. This study represents a significant progress in the characterization of the genetic regulation of the main immune cell type involved in the pathogenesis of RA.

## Supporting Information

Table S1 **Main epidemiological and clinical features of the RA patients included in this study.** Abbreviations: DAS28, Disease Activity Score.
(DOC)

Table S2 **Total genes used in the comparative eQTL analysis between RA CD4+ T cells and LCLs.**
(XLS)

Table S3 ***Cis*** **-1Mb associations obtained in the genome-wide** ***cis*** **-eQTL analysis in RA CD4+ T cells (P_FDR_<0.5).** Abbreviations: Gene chr, Chromosomal location of the expressed gene; Gene coord, Coordinates of the expressed gene; Bp, Base pair; SNP chr, Chromosomal location of the SNP; SNP coord, Coordinates of the SNP; FDR, P-value False Discovery Rate.
(XLS)

Table S4 ***Trans*** **associations in CD4+ T cells for M9 module genes (P<1e-5).** Abbreviations: Gene chr, Chromosomal location of the expressed gene; Gene coord, Coordinates of the expressed gene; Bp, Base pair; SNP chr, Chromosomal location of the SNP; SNP coord, Coordinates of the SNP; Beta, Slope coefficient; FDR, P-value False Discovery Rate.
(XLS)

Table S5 ***Trans*** **associations in CD4+ T cells for M12 module genes (P<1e-5).** Abbreviations: Gene chr, Chromosomal location of the expressed gene; Gene coord, Coordinates of the expressed gene; Bp, Base pair; SNP chr, Chromosomal location of the SNP; SNP coord, Coordinates of the SNP; Beta, Slope coefficient; FDR, P-value False Discovery Rate.
(XLS)

Table S6 ***Cis*** **-1Mb associations in CD4+ T cells (P<5e-2) that were used to characterize the gene regulatory profiles.** Abbreviations: Gene chr, Chromosomal location of the expressed gene; Gene coord, Coordinates of the expressed gene; Bp, Base pair; SNP chr, Chromosomal location of the SNP; SNP coord, Coordinates of the SNP; Beta, Slope coefficient; FDR, P-value False Discovery Rate.
(XLS)

Table S7 ***Trans*** **associations in CD4+ T cells (P<1e-5) that were used to characterize the gene regulatory profiles.** Abbreviations: Gene chr, Chromosomal location of the expressed gene; Gene coord, Coordinates of the expressed gene; Bp, Base pair; SNP chr, Chromosomal location of the SNP; SNP coord, Coordinates of the SNP; Beta, Slope coefficient; FDR, P-value False Discovery Rate.
(XLS)

Table S8 ***Cis*** **-1Mb associations in LCLs (P<5e-2) that were used to characterize the gene regulatory profiles.** Abbreviations: Gene chr, Chromosomal location of the expressed gene; Gene coord, Coordinates of the expressed gene; Bp, Base pair; SNP chr, Chromosomal location of the SNP; SNP coord, Coordinates of the SNP; Beta, Slope coefficient; FDR, P-value False Discovery Rate.
(XLS)

Table S9 ***Trans*** **associations in LCLs (P<1e-5) that were used to characterize the gene regulatory profiles.** Abbreviations: Gene chr, Chromosomal location of the expressed gene; Gene coord, Coordinates of the expressed gene; Bp, Base pair; SNP chr, Chromosomal location of the SNP; SNP coord, Coordinates of the SNP; Beta, Slope coefficient; FDR, P-value False Discovery Rate.
(XLS)
