# Supplementary material for: Novel Insights into the Regulatory Architecture of CD4+ T Cells in Rheumatoid Arthritis
Source: PLoS One. 2014 Jun 24;9(6):e100690. doi: 10.1371/journal.pone.0100690 (PMC4069080; doi:10.1371/journal.pone.0100690)
Supplement: Table S1 — Main epidemiological and clinical features of the RA patients included in this study. Abbreviations: DAS28, Disease Activity Score. (DOC) [file pone.0100690.s001.doc]

**Table S1. Main epidemiological and clinical features of the RA patients included in this study.**

| **Patients** | **Age (years)** | **Gender** | **DAS28 (score)** |
| --- | --- | --- | --- |
| RA patient 1 | 50 | Female | 5.40 |
| RA patient 2 | 58 | Female | 6.30 |
| RA patient 3 | 42 | Female | 4.10 |
| RA patient 4 | 69 | Female | 5.20 |
| RA patient 5 | 34 | Female | 5.50 |
| RA patient 6 | 49 | Female | 3.50 |
| RA patient 7 | 58 | Female | 5.90 |
| RA patient 8 | 60 | Female | 5.60 |
| RA patient 9 | 61 | Female | 6.00 |
| RA patient 10 | 67 | Female | 6.60 |
| RA patient 11 | 58 | Female | 5.90 |
| RA patient 12 | 61 | Female | 7.20 |
| RA patient 13 | 68 | Male | 5.60 |
| RA patient 14 | 48 | Male | 4.50 |
| RA patient 15 | 69 | Female | 6.20 |
| RA patient 16 | 48 | Female | 5.30 |
| RA patient 17 | 60 | Female | 6.80 |
| RA patient 18 | 53 | Male | 5.90 |
| RA patient 19 | 49 | Female | 6.20 |
| RA patient 20 | 57 | Female | 7.60 |
| RA patient 21 | 47 | Female | 7.10 |
| RA patient 22 | 57 | Female | 5.00 |
| RA patient 23 | 44 | Female | 6.26 |
| RA patient 24 | 53 | Female | 7.59 |
| RA patient 25 | 68 | Female | 8.00 |
| RA patient 26 | 44 | Female | 6.67 |
| RA patient 27 | 33 | Female | 7.43 |
| RA patient 28 | 67 | Female | 8.32 |
| RA patient 29 | 59 | Female | 7.37 |
| **Summary** | 54.86 +/- 9.9 | 90% female | 6.17 +/- 1.15 |
